# Development and validation of a RNA binding protein gene pair-associated prognostic signature for prediction of overall survival in hepatocellular carcinoma

**DOI:** 10.1186/s12938-020-00812-0

**Published:** 2020-09-01

**Authors:** Chunmiao Kang, Xuanhui Jia, Hongsheng Liu

**Affiliations:** 1 Department of Ultrasound, Shaanxi Provincial People’s Hospital, Xi’an, 710068 China; 2 Department of Radiology, Xi’an Central Hospital Affiliated to Xi’an Jiaotong University, No. 161, Xiwu Road, Xincheng District, Xi’an, 710003 Shaanxi PR China

**Keywords:** Hepatocellular carcinoma, RNA binding protein, Signature, Overall survival

## Abstract

**Background:**

Increasing evidence has demonstrated the correlation between hepatocellular carcinoma (HCC) prognosis and RNA binding proteins (RBPs) dysregulation. Thus, we aimed to develop and validate a reliable prognostic signature that can estimate the prognosis for HCC.

**Methods:**

Gene expression profiling and clinical information of 374 HCC patients were derived from the TCGA data portal. The survival-related RBP pairs were determined using univariate cox-regression analysis and the signature was built based on LASSO analysis. All patients were divided patients into high-and low-risk groups according to the optimal cut off of the signature score determined by time-dependent receiver operating characteristic (ROC) curve analysis. The predictive value of the signature was further validated in an independent cohort.

**Results:**

A 37-RBP pairs signature consisting of 61 unique genes was constructed which was significantly associated with the survival. The RBP-related signature accurately predicted the prognosis of HCC patients, and patients in high-risk groups showed poor survival in two cohorts. The novel signature was an independent prognostic factor of HCC in two cohorts (all P < 0.001). Furthermore, the C-index of the prognostic model was 0.799, which was higher than that of many established risk models. Pathway and process enrichment analysis showed that the 61 unique genes were mainly enriched in translation, ncRNA metabolic process, RNA splicing, RNA modification, and translational termination.

**Conclusion:**

The novel proposed RBP-related signature based on relative expression orderings could serve as a promising independent prognostic biomarker for patients with HCC, and could improve the individualized survival prediction in HCC.

## Background

Hepatocellular carcinoma (HCC) was the most common primary malignancy of the liver and its incidence rate is increasing [1]. It has been commonly known that many risk factors contribute to HCC carcinogenesis and progression, including chronic hepatitis B virus (HBV)/hepatitis C virus (HCV) infection, diabetes mellitus, alcohol abuse, obesity, nonalcoholic fatty liver disease, metabolic diseases, autoimmune hepatitis, and exposure to dietary toxins such as aflatoxins [2]. Recently, it has reported that the incidence of HCC all over the world is highly heterogeneous owe to different risk factors, and that most HCC patients occur in South-eastern Asia and Saharan Africa, where HBV infection is the leading risk factor [3]. Despite the rapid progress over the past few decades in earlier diagnosis and treatment of HCC, the long-term prognosis remains poor, and the 5-year survival rate remains below 20% [4]. Surgery resection remains the most effective treatment of HCC, and it has markedly improved the overall survival (OS) of HCC patients. However, the long-term survival rate is still low [4, 5]. The classic tumor-node-metastasis (TNM) staging, as a classic prognostic model, helps predict HCC prognosis and is widely used in current clinical practice [6]. However, the predictive efficacy of TNM model is still far from satisfying. HCC is a highly heterogeneous malignancy with substantially variable clinical outcomes, the prognoses of patients with the same TNM stage may varied due to inherent clinical and molecular diversities, and even among patients with HCC who are diagnosed as the same TNM stage and received similar clinical management, survival outcomes are various, suggesting that TNM provides incomplete prognosis information [7, 8]. Therefore, novel valid and robust prognostic signatures are indispensable to improve risk prediction and offer better information for guiding personalized therapy.

RNA-binding proteins (RBPs) are inherently pleiotropic proteins, which bind RNA through one or more spherical RNA-binding domains and control RNA stabilization, degradation and modification at the post-transcriptional level [9]. So far, more than 1,500 RBP genes have been confirmed by genome-wide screening in human genome [10]. Emerging evidences have demonstrated that RBPs are critical in the regulation of human cancer progression by influencing multifaceted cellular functions [11, 12]. RBPs play a vital role in post-transcription and dysfunction of RBPs expression is closely related to various diseases including cancer and immune disorders [13]. Considering the importance of post-transcriptional regulation of RBPs, aberrantly deregulated RBPs are closely related to the occurrence and progression of cancers. To best of our knowledge, very few RBP based signatures have been established for HCC prognosis prediction.

With the rapid development of sequencing and precision medicine, increasing evidence indicated that gene signatures at mRNA level had promising potential in predicting HCC prognosis. Numerous studies have proposed various signatures for survival prediction in patients with HCC [14–19]. For example, Liu et al. [20] have established a novel six-gene signature for HCC prognosis prediction, but only one external validation cohort was used to validate the performance of the predicted model without comparison of its performance with other existed biomarkers. Li et al. developed and identified a seven-gene signature related to the DNA repair process to predict survival in HCC, but without any external validation the performance of the predicted model [21]. However, none of these signatures have yet been widely accepted in routine clinical practice because of technical limitations and evaluation difficulties. Recently, a novel established method based on the within-sample relative expression orderings of gene pairs has been proposed. It can overcome the disadvantages of gene expression data normalization and scaling, and has yielded robust results in various studies [22, 23].

Therefore, based on 1542 RBP genes [10], we used two cohorts to develop and validate an individualized prognostic signature for HCC. We compared this signature with other previous prognostic signatures to validate the predictive effectiveness and accuracy of the novel signature.

## Results

### Prognostic signature construction

**Fig. 1:**
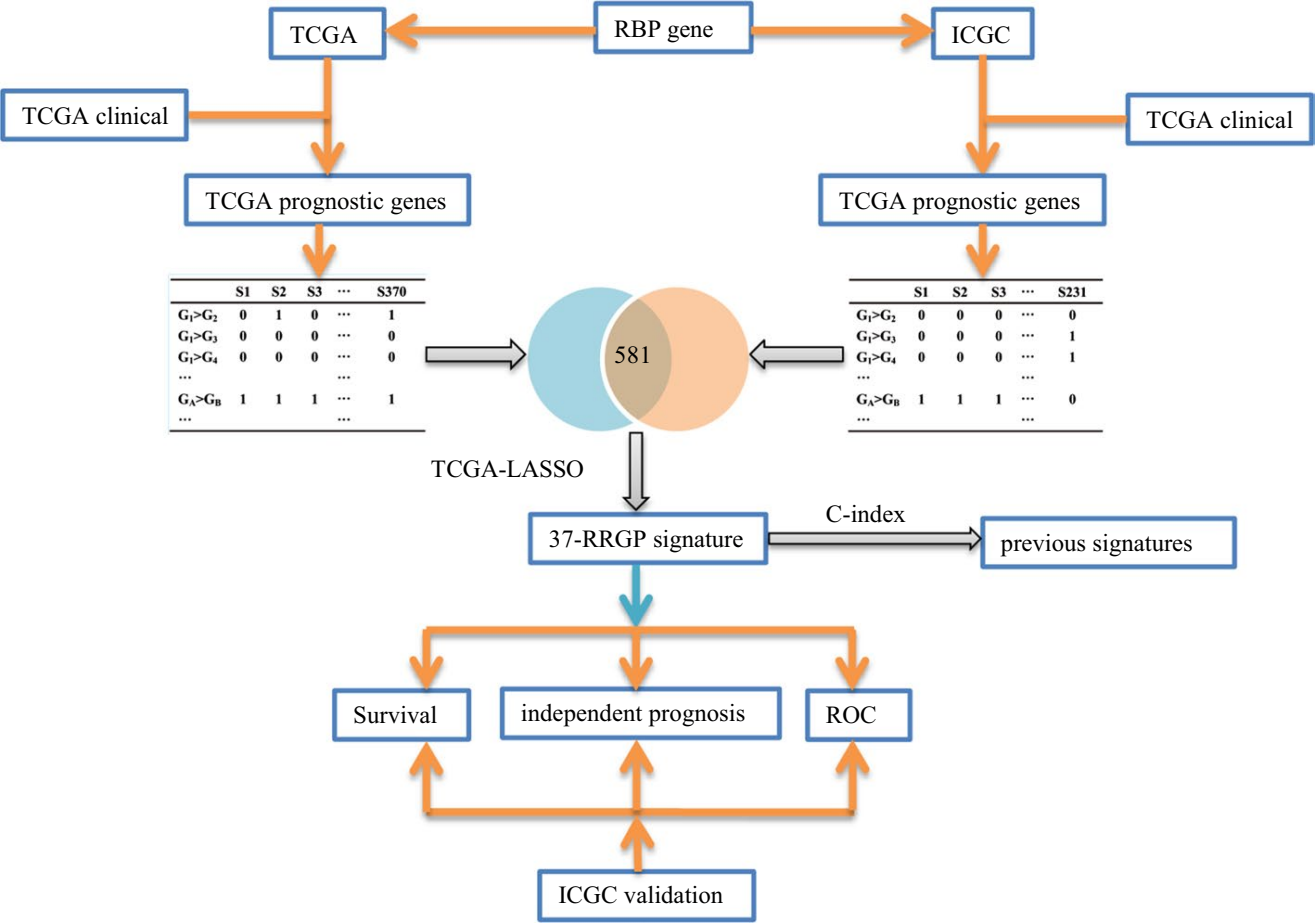
Analysis workflow used in this study

**Fig. 2:**
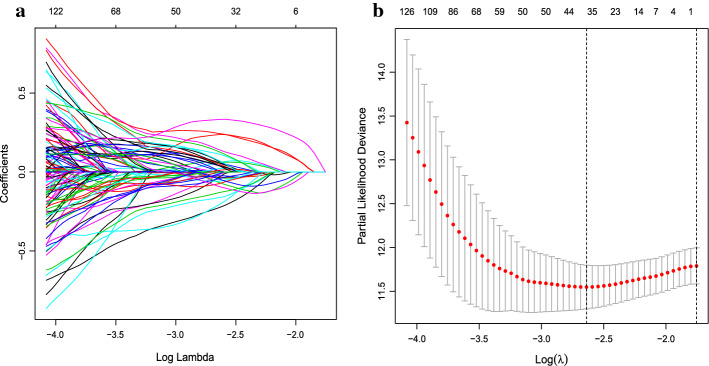
Predictor selection by the least absolute shrinkage and selection operator (LASSO). **a** Parameter (Lambda) selection by LASSO model adopted tenfold cross-validation via minimum criteria; **b** LASSO coefficient profile plot of 37 RBP gene pairs against the log (Lambda) sequence

The analysis process of present study is shown in Fig. 1. A total of 647 RBP genes were common among two datasets, and 23,353 RRGPs were constructed in two cohorts. Using univariate Cox regression analysis, we identified 581 prognostic RRGPs that were significantly associated with patient OS (P < 0.05). To determine the optimal model for predicting prognosis, the prognostic RRGPs were used to build prognostic signature by using LASSO penalized Cox regression on the TCGA cohort. The risk score was calculated as combination of gene’ expression values weighted by regression coefficient derived from LASSO regression. After 1000 iterations, we identified the 37 gene pairs to construct an RRGP risk signature (Fig. 2). The 37-RRGP prognostic signature information is shown in Table 1.

**Table 1:** 37-RRGP prognostic signature information

Genepair1	Gene name	Genepair1	Gene name	Coefficient
RBFOX2	RNA binding fox-1 homolog 2	TRMT6	tRNA methyltransferase 6	− 0.03268748
RBFOX2	RNA binding fox-1 homolog 2	UPF3B	UPF3B regulator of nonsense mediated mRNA decay	− 0.037164549
NCBP2	Nuclear cap binding protein subunit 2	AIMP1	Aminoacyl tRNA synthetase complex interacting multifunctional protein 1	0.066221909
GNL2	G protein nucleolar 2	QTRT1	Queuine tRNA-ribosyltransferase catalytic subunit 1	0.059558561
PELO	Pelota mRNA surveillance and ribosome rescue factor	DHX34	DExH-box helicase 34	− 0.016014421
FBXO17	F-box protein 17	ISG20L2	Interferon stimulated exonuclease gene 20 like 2	− 0.161981498
FBXO17	F-box protein 17	YARS	Tyrosyl-tRNA synthetase	− 0.032387367
SPATS2	Spermatogenesis associated serine rich 2	DHX58	DExH-box helicase 58	0.236866919
LSM4	LSM4 homolog, U6 small nuclear RNA and mRNA degradation associated	CIRBP	Cold inducible RNA binding protein	0.05881972
PIH1D1	PIH1 domain containing 1	SRRT	Serrate, RNA effector molecule	− 0.225462618
MRPL54	Mitochondrial ribosomal protein L54	PA2G4	Proliferation-associated 2G4	− 0.067034961
MRPL54	Mitochondrial ribosomal protein L54	SMG5	SMG5 nonsense mediated mRNA decay factor	− 0.024001874
HINT3	Histidine triad nucleotide binding protein 3	ABCE1	ATP binding cassette subfamily E member 1	− 0.206441474
RPS19BP1	Ribosomal protein S19 binding protein 1	ACO1	Aconitase 1	0.062394557
MRPS28	Mitochondrial ribosomal protein S28	TXNL4A	Thioredoxin like 4A	− 0.058273164
RPUSD1	RNA pseudouridine synthase domain containing 1	ZC3H13	Zinc finger CCCH-type containing 13	0.140504191
SARS	Seryl-tRNA synthetase	MRPL40	Mitochondrial ribosomal protein L40	0.004986247
ANG	Angiogenin	RPL15	Ribosomal protein L15	− 0.005968985
XPOT	Exportin for tRNA	PRPF8	Pre-mRNA processing factor 8	0.057395002
DHX34	DExH-box helicase 34	SRPK2	SRSF protein kinase 2	0.038955695
DHX34	DExH-box helicase 34	DDX59	DEAD-box helicase 59	0.021534239
EZH2	Enhancer of zeste 2 polycomb repressive complex 2 subunit	PPRC1	PPARG related coactivator 1	0.049244644
KHDRBS3	KH RNA binding domain containing, signal transduction associated 3	PPARGC1A	PPARG coactivator 1 alpha	0.243268497
MTRF1	Mitochondrial translation release factor 1	CNOT6	CCR4-NOT transcription complex subunit 6	− 0.041893707
ZC3H13	Zinc finger CCCH-type containing 13	ESF1	SF1 nucleolar pre-rRNA processing protein homolog	− 0.073576247
UPF3B	UPF3B regulator of nonsense mediated mRNA decay	DDX59	DEAD-box helicase 59	0.005316586
VARS2	Valyl-tRNA synthetase 2, mitochondrial	EEF1E1	Eukaryotic translation elongation factor 1 epsilon 1	− 0.092573503
PPIH	Peptidylprolyl isomerase H	AIMP1	Aminoacyl tRNA synthetase complex interacting multifunctional protein 1	0.011338774
AARS	Alanyl-tRNA synthetase	SF3B4	Splicing factor 3b subunit 4	− 0.130223697
CTIF	Cap binding complex dependent translation initiation factor	RRP12	Ribosomal RNA processing 12 homolog	− 0.011438504
TRMT1	tRNA methyltransferase 1	SRA1	Steroid receptor RNA activator 1	0.113049796
DENR	Density regulated re-initiation and release factor	PRPF8	pre-mRNA processing factor 8	0.040880372
NPM1	Nucleophosmin 1	RPL9	Ribosomal protein L9	0.127539928
ZFC3H1	Zinc finger C3H1-type containing	DDX60	DExD/H-box helicase 60	0.039777048
MRPS31	Mitochondrial ribosomal protein S31	REXO4	REX4 homolog, 3′-5′ exonuclease	− 0.045609959
SMG5	SMG5 nonsense mediated mRNA decay factor	NOL7	Nucleolar protein 7	0.07179385
YARS	Tyrosyl-tRNA synthetase	SRA1	Steroid receptor RNA activator 1	0.33277612

### Validation and assessment of the novel signature

**Fig. 3:**
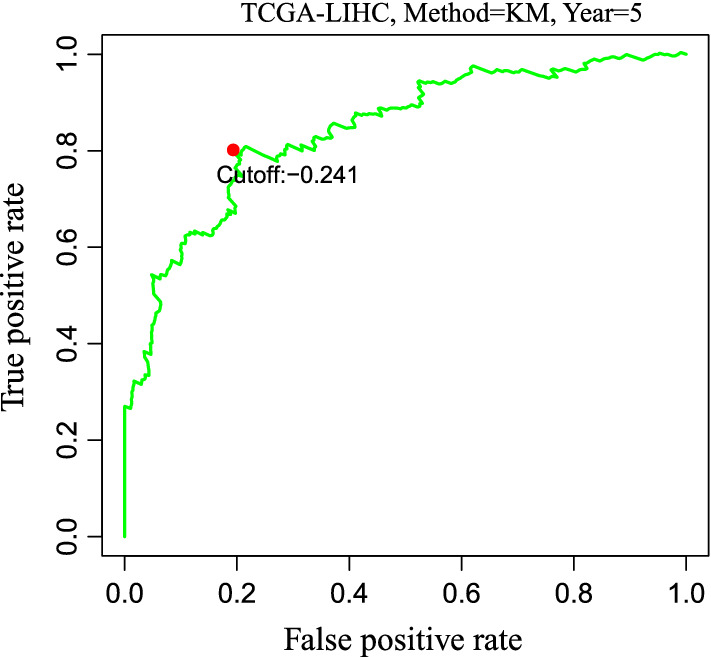
Time-dependent ROC curve for the signature risk score in the TCGA cohort. The risk score of -0.241 was used as cut-off to separate patients into low- and high-risk groups

**Fig. 4:**
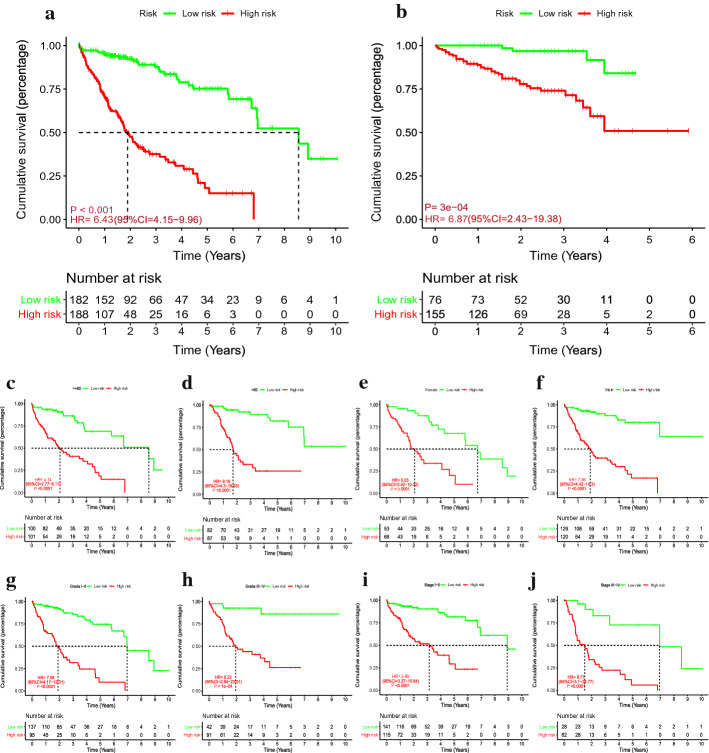
Kaplan–Meier survival analysis of patients in the high-risk and low-risk groups in the TCGA cohort (**a**), and ICGC dataset (**b**). The Kaplan–Meier curves of patients with hepatocellular carcinoma stratified by age (**c**, **d**), sex (**e**, **f**), grade (**g**, **h**), and stage (**i**, **j**)

**Fig. 5:**
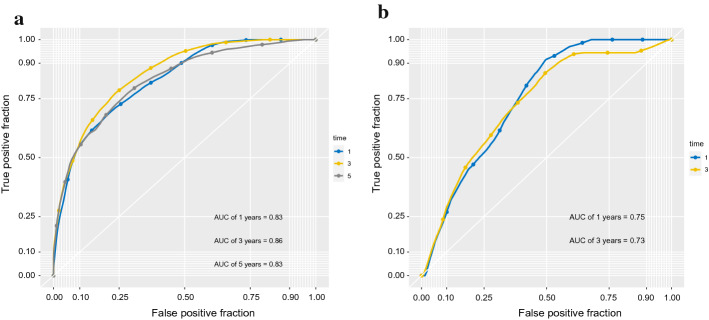
The time-dependent ROC curves for the signature predicting 1-, 3- and 5-year overall survival of hepatocellular carcinoma in the TCGA cohort (**a**), and the external cohort (**b**)

**Fig. 6:**
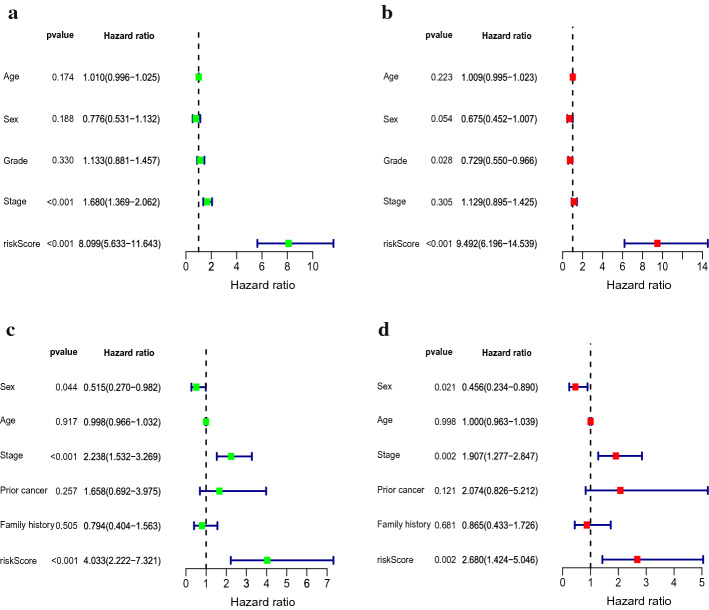
Univariate and multivariate analyses to identify independent prognostic variables for OS of HCC in the TCGA cohort (**a**, **b**), and external cohort (**c**, **d**)

The clinical data of patients in the internal validation group and the external validation group is displayed in Table 2. We calculated the risk score for each patient as mentioned previously in the two groups. The optimal cut-off of the signature risk score for separating patients into the high- or low-risk groups was set at -0.241 using time-dependent ROC curve analysis at 5 years (Fig. 3). It was revealed that the high-risk group presented a poorer OS than the low-risk group (P < 0.001; HR = 6.43, 95%CI 4.15–9.96) (Fig. 4a). The patients in the ICGC cohort were classified into high- and low-risk groups based on the same cutoff value in the TCGA cohort. It was confirmed that high-risk group exhibited a poorer OS than the low-risk group (P < 0.001; HR = 6.87, 95%CI 2.43–19.38) (Fig. 4b). The Kaplan–Meier curves indicated that the risk score was a stable prognostic marker for patients with HCC stratified by age (< 60 or ≥ 60), sex (male or female), stage (I-II or III-IV), and grade (I-II or III-IV) (Fig. 4c–j). Furthermore, the AUC values of the prognostic signature risk score for the 1-year, 3-year and 5-year OS using the time-dependent ROC curves in the TCGA cohort were 0.83, 0.86, and 0.83, respectively (Fig. 5a). As expected, the robust predictive performance of the signature was further validated in the ICGC cohort with the 1-, and 3-year survival rates of 0.75 and 0.73, respectively, (Fig. 5b), which demonstrated that the predictive ability of our prognostic signature was robust and accurately. To further explore the prognostic power of the signature for other clinical factors, univariate and multivariate Cox proportional hazards regression analyses were used to the TCGA cohort. The univariate and multivariate Cox regression analysis showed that the novel signature risk score was an independent prognostic factor for predicting OS of HCC after adjustment for by age, gender, grade, and stage in the TCGA cohort (HR = 9.492, 95%CI 6.196–14.539; P < 0.001, Fig. 6a, b), and further confirmed in the external cohort (HR = 2.680, 95%CI 1.424–5.046, P = 0.002, Fig. 6c–d).

**Table 2:** Clinical data of patients in the internal validation group and the external validation group

Variables	Subgroups	TCGA(N = 370)	ICGC(N = 231)
Age			
	< 60	169	44
	≥ 60	201	187
Sex			
	Male	249	170
	Female	121	61
Stage			
	I	171	36
	II	85	105
	III	85	71
	IV	5	19
	NA	24	0
Grade			
	I	55	–
	II	177	–
	III	121	–
	IV	12	–
	NA	5	–
Survival status			
	Dead	130	42
	Living	240	189
Family history			
	Positive	–	73
	Negative	–	143
	NA	–	15
Prior malignancy			
	Positive	–	30
	Negative	–	201
	NA	–	0

### Comparison with other established prognostic signatures

**Fig. 7:**
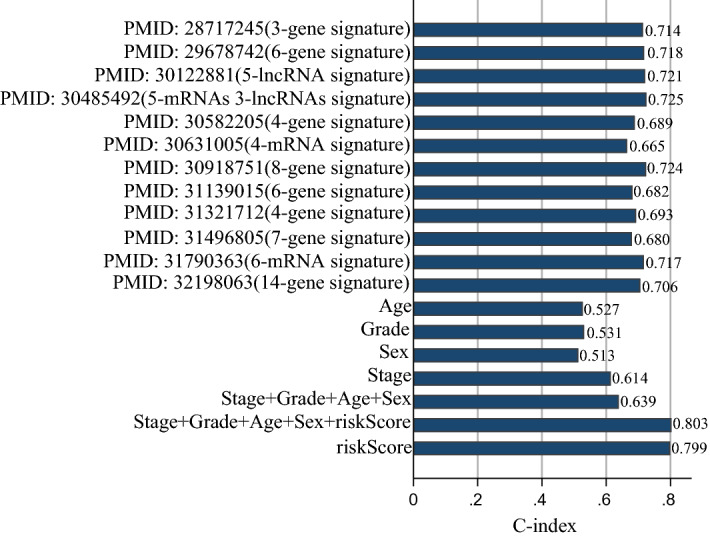
Comparison of C-index among multivariate prognostic modules, signature risk score, twelve existing prognostic signatures and clinical features

To further confirm the prognostic performance of the model, we built prognostic risk models of age, sex, stage and grade and compared these models with the novel signature risk score. The 37-RRGP signature achieved a higher predictive accuracy than the risk models of age, sex, grade, stage, and their combined models with C-index of 0.799 (Fig. 7). The RBP prognostic signature was verified as a robust complement to clinical and pathological factors for the prognosis assessment of HCC patients with C-index of 0.803. We further compared the novel established signature with twelve published molecular signatures [16–21, 24–29], which were all prognostic signatures for HCC survival prediction. More importantly, the C-index of the prognostic model yielded much higher value than that of other risk models (Fig. 7). Thus, the novel prognostic signature was more effective than the previous signatures in the prognosis of HCC patients.

### Biological processes related to the RBP signature

**Fig. 8:**
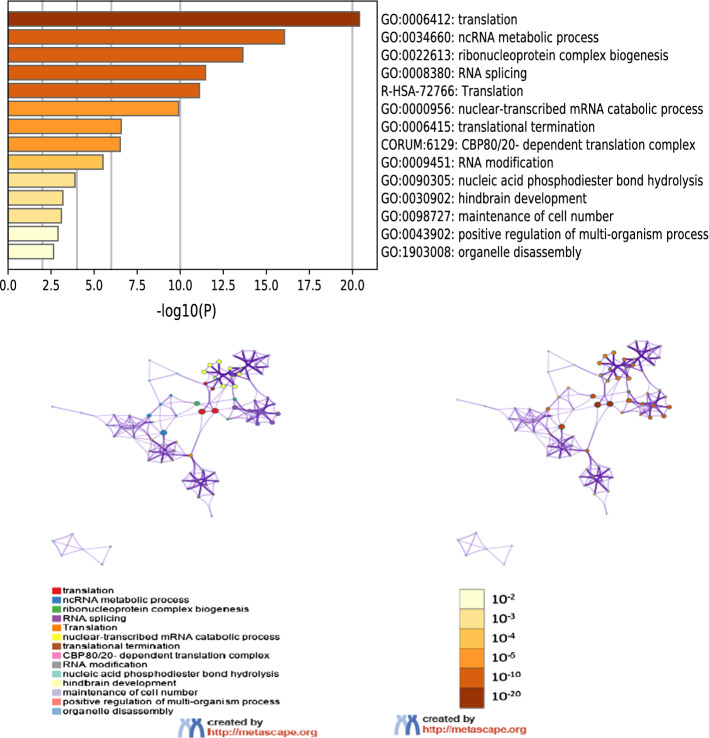
Functional enrichment of 61 unique RBP-related genes using the Metascape database

Functional enrichment of the RBP genes relevant to the signature in the TCGA cohort were mostly involved in RNA modification, translation, translational termination, nuclear-transcribed mRNA catabolic process, RNA splicing, ribonucleoprotein complex biogenesis, and ncRNA metabolic process (Fig. 8). The enrichment of related biological processes provided evidence of molecular mechanisms affected by the RBP signature and, therefore, contributed to predict the prognosis of HCC.

## Discussion

HCC is a heterogeneous solid malignancies and the prognosis of HCC mainly depends on the degree of surgical resection and intervention [18]. Therefore, it’s important to accurate predict survival and adopt novel therapy methods in time for the patients at high risk of mortality. Microarray technology, which is a highly efficient and accurate transcriptional expression technology, has been successfully used in the identifying of molecular biomarkers of nearly all human malignant cancers. With the rapid developments in gene chips as well as high-throughput sequencing, gene signatures based on mRNA expression levels have exhibited significant potential in predicting HCC prognosis. Many risk-coefficient models based on a multigene mRNA expression signature has been reported and confirmed to be an independent prognostic predictor for OS in HCC and could classify patients into high- and low-risk group with notably different OS [14, 18–21, 24, 27]. Although a lot of multigene prognostic signatures have been developed for HCC patients, but the accuracy of their prognosis predictions remains far from satisfying [14–18, 24, 25]. Therefore, there is an urgent to build robust prognostic signatures to predict the survival of patients with HCC.

In the last years, post-transcriptional regulation of gene expression has stimulated much interest in studies focused on RBPs and the interactions with their RNA targets [30]. Previous studies have demonstrated that RBPs were abnormally expressed in different cancer types, which affected the translation of mRNA into protein, and were indeed involved in carcinogenesis [31]. For example, SERBP1 (Serpine1 mRNA-binding protein 1) is a member of the RG/RGG family of RNA-binding proteins and has been recognized as a novel oncogenic factor in glioblastoma, and the high SERBP1 expression correlates with poor patient survival and adverse response to chemo- and radiotherapy [32]. RNA-binding protein CELF1 contributes to migration, invasion, and chemotherapy resistance by targeting ETS2 in colorectal cancer and could be a potential diagnostic and prognostic marker [33]. However, little attention has been paid to the molecular characteristics of RBP genes interaction in HCC. Therefore, there is a need to develop and validate of a robust RBP genes signature for prediction of the prognosis of HCC.

In this study, we used the within-sample relative expression orderings to develop a signature based on 37-RRGP for HCC patients and validated its accuracy and effectiveness in an independent cohort via comprehensive bioinformatics methods. Univariate Cox regression analysis was used to identify RRGPs significantly associated with OS in HCC. We found that 581 gene pairs were associated with OS, with p < 0.05. We identified the 37 RRGPs that predicted survival in patients with a LASSO penalized Cox regression on the TCGA cohort. These gene pairs were used to derive a signature risk score. Based on the cutoff of the risk score, patients could effectively classify into low-risk and high-risk groups with distinct outcomes. Furthermore, the same conclusion was reached in survival analysis stratified by age, sex, stage, and grade. We performed univariate and multivariate Cox regression analysis to investigate the combined ability of the signature risk score and other clinicopathological factors to predict survival. It was revealed that the novel signature risk score may be an independent prognostic factor for patients with HCC. Besides, an independent dataset from ICGC was used as validation set to ensure the robustness of our results. The results confirmed that the risk score is a stable, independent prognostic indicator and indicates that the risk score could be of important significance for patients with HCC as an effective clinical classification tool. However, most of existing signatures have not been widely used in clinical practice, which may owe to multiple factors. First, many traditional prognostic signatures failed to validate their findings in another independent cohort [17, 21]. More importantly, the diversity of data also represents a major challenge, and data processing across various sequencing platforms require suitable normalization and elimination of the batch effects. In addition, the cutoff in previous signatures could not be used across multiple datasets. These disadvantages severely limit their clinical application. In this study, we performed gene pairwise analysis to identify reliable biomarkers for prognosis of HCC based on the within-sample relative expression orderings of genes, which are robust against to experimental batch effects [34, 35]. Using this algorithm, the bias caused by gene normalization was eliminated. Furthermore, the signature and cutoff value could be used across different datasets, which was an important advantage in our study. In addition, the RBP-based prognostic signature identified in the present study performed well compared with twelve existing prognostic signatures, which was another vital advantage in our study. Furthermore, the time-dependent ROC analysis showed that it performed well in 1-, 3, and 5 years for HCC OS prediction. All these results demonstrated that the novel signature could provide an accurate prognosis of patients with HCC.

The RBP genes involved in the signature were mainly involved in RNA modification, translation, translational termination, RNA splicing, nuclear-transcribed mRNA catabolic process, ribonucleoprotein complex biogenesis, and ncRNA metabolic process. Previous studies revealed that RBPs can bind to their target RNAs in a structure or sequence-dependent manner to form ribonucleoprotein complexes that regulate processes ranging from mRNA stability to RNA processing, splicing, localization, export, as well as translation at the post-transcriptional level [36]. Post-transcriptional regulation by non-coding RNAs has been reported involved in RBP expression in cancer. For instance, the expression of HuR protein is antagonized by miR-519 and miR-125a, remarkably inhibiting the proliferation of colon, cervical, breast, and ovarian carcinoma cells [37, 38]. It was revealed that ribonucleoprotein granule is a crucial region that executes protein biosynthesis. The alteration of ribonucleoprotein influences the translation processing and related to cancer progression [39]. RNA binding protein SERBP1, which regulates mRNA translation, has been identified as a target of the tumor suppressor miR-218 in HCC and confirmed associated with cell migration/invasion and epithelial mesenchymal transition [40]. A recent study revealed that methyltransferase-like 3 (METTL3) suppressed the expression of morphological effect on genitalia 1 (SMG1) through m6A modification-mediated miR-873-5p up-regulation, therefore, serving an oncogenic role in HCC [41]. The number of HCC cells is significantly reduced when RNA binding protein NSUN6 is overexpressed, revealing that the occurrence and development of LIHC is related to alterations of tRNACys and tRNAThr biogenesis [42]. These evidences suggested that the signature may be concerned with HCC-related biological pathways and their functional dysregulations may be closely related to the survival of HCC.

Nevertheless, some notable limitations must be acknowledged. The main limitation of our findings is its retrospective nature, and more prospective studies should be conducted to validate the findings. Moreover, the underlying mechanism through which the identified RBP genes contribute to the initiation and progression of HCC still requires further evaluated using RT-PCR or IHC.

## Conclusion

We developed and validated of a novel robust prognostic signature based on relative RBP genes expression orderings, which could serve as a promising independent prognostic biomarker for patients with HCC. The novel signature could improve the individualized outcome prediction in HCC. The RBP-related signature provided new insights into the identification of HCC patients with a high risk of mortality.

## Materials and methods

### HCC data sources and data preprocessing

Level-three transcriptome RNA-sequencing data (HTSeq-FPKM) of 374 primary HCC samples, as well as their clinical follow-up information were downloaded from the TCGA data portal (https://cancergenome.nih.gov/). Another RNA-seq dataset of 240 primary HCC patients together with corresponding clinical information were accessed from the International Cancer Genome Consortium database (ICGC, https://dcc.icgc.org/, LIRI-JP), which was used as cohort for external validation of the signature. For the TCGA cohort, the expression values at probe level (probe ID) were converted to the corresponding gene symbol according to the annotation files without further standardization. When several probes matched to an identical gene symbol, the mean value was calculated as the expression value of this gene. We matched the ID numbers of the patients with their corresponding mRNA expression profile and clinical data and excluded patients whose ID numbers failed to match. Only selected patients with complete overall survival (OS) information were used for further analysis. We downloaded 1542 RBP genes as mentioned above. The RBP genes expression matrixes were further extracted from the two publicly datasets, respectively.

### Prognostic signature construction

Before formal statistical analysis, we first filtered out RBPs measured on all the platforms with relatively high variation (determined by median absolute deviation > 0.5) to decrease the false discoveries [43, 44]. The gene expression levels were compared pairwise in a given sample or profile to compute a score for each RBP-related gene pair (RRGP). In a pairwise comparison, if the expression value of the first RBP gene was higher than that of the second one, the output score of this RRGP was 1; otherwise, the output was 0, according to the proposed method [15, 22]. The advantage of analyzing genes in a pairwise comparison is without the requirement for standardization process for individualized analysis. A summary of 23,353 shared RRGPs in two datasets were included as the initial candidate factors for prognosis prediction. Univariate analysis was performed to identify prognostic RRGPs associated with OS (P < 0.05). The RRGPs was further reduced while maintaining high accuracy. Therefore, LASSO penalized Cox regression was used to establish a more stable prognostic signature using an R package glmnet after 1000 iterations with tenfold cross-validation. Subsequently, the risk score of the prognostic RBP signature for each sample was calculated by the relative expression level of RRGPs with weighted by the estimated regression coefficient derived from the LASSO regression model. *Risk score* = *(Exprgenepair-1* × *Coefgenepair-1)* + *(Exprgenepair-2* × *Coefgenepair-2)* + *…* + *(Exprgenepair-n* × *Coefgenepair-n).* The patients were divided into high-and low-risk groups according to the RRGPs score cutoff, which was determined by a time-dependent ROC curve at 5 years.

### Validation and assessment of the novel signature

We adopted the nearest neighbor estimation (NNE) method to plot the ROC curve. Time-dependent ROC with AUC at 1, 3, and 5 years was used to explore the prognostic accuracy in both cohorts. The OS difference between the low-risk and high-risk groups were evaluated by the log-rank test and Cox regression analysis. We then integrated the signature risk score with existing clinical and pathologic variables for multivariate Cox regression analysis. The Kaplan–Meier survival curves in patients with HCC stratified by sex, age, grade, and stage were used to further validate the performance of the prognostic signature. In addition, we compared the prognostic signature with other clinicopathological features and twelve recent established prognostic signatures by the Harrell’s concordance index (C-index) with 1,000 bootstraps resamples in the TCGA cohort.

### Functional enrichment of the RBP genes in the prognostic signature

To understand the underlying biological mechanisms of the novel RBP-related prognostic signature, functional enrichment analysis was conducted among the 61 unique genes using the Metascape database, which is a biologist-oriented resource for the analysis of systems-level datasets [45].

### Statistical analysis

Survival curves were generated using the Kaplan–Meier method and the differences in survival curves were compared by the log-rank test using the ‘survival’ package. Multivariate analyses were conducted using the Cox proportional hazards regression model and hazard ratios (HR) with their 95% confidence interval (CI) were calculated. The ROC curves were performed by an R package “survivalROC”. The R package rms was used to compare other models with the RRGP prognostic signature. A p-value < 0.05 was considered to be significant. All statistical analyses were performed using R (version 3.6.3; https://www.r-project.org/).

## Data Availability

The raw data of this study are derived from the TCGA database (https://portal.gdc.cancer.gov/) and ICGC data portal (https://dcc.icgc.org/), which are publicly available databases.

## References

[1] El-Serag HB (2011). Hepatocellular carcinoma. N Engl J Med.

[2] Yang JD, Kim WR, Coelho R, Mettler TA, Benson JT, Sanderson SO, Therneau TM, Kim B, Roberts LR (2011). Cirrhosis is present in most patients with hepatitis B and hepatocellular carcinoma. Clin Gastroenterol Hepatol.

[3] Goh GB, Chang PE, Tan CK (2015). Changing epidemiology of hepatocellular carcinoma in Asia. Best Pract Res Clin Gastroenterol.

[4] Allemani C, Weir HK, Carreira H, Harewood R, Spika D, Wang XS, Bannon F, Ahn JV, Johnson CJ, Bonaventure A (2015). Global surveillance of cancer survival 1995–2009: analysis of individual data for 25,676,887 patients from 279 population-based registries in 67 countries (CONCORD-2). Lancet (London, England).

[5] Kamo N, Kaido T, Yagi S, Okajima H, Uemoto S (2018). Liver transplantation for intermediate-stage hepatocellular carcinoma. Liver cancer.

[6] Roayaie S, Blume IN, Thung SN, Guido M, Fiel MI, Hiotis S, Labow DM, Llovet JM, Schwartz ME (2009). A system of classifying microvascular invasion to predict outcome after resection in patients with hepatocellular carcinoma. Gastroenterology.

[7] Bruix J, Gores GJ, Mazzaferro V (2014). Hepatocellular carcinoma: clinical frontiers and perspectives. Gut.

[8] Dhir M, Melin AA, Douaiher J, Lin C, Zhen WK, Hussain SM, Geschwind JF, Doyle MB, Abou-Alfa GK, Are C (2016). A review and update of treatment options and controversies in the management of hepatocellular carcinoma. Ann Surg.

[9] Scaturrok M, Sala A, Cutrona G, Raimondi L, Cannino G, Fontana S, Pucci-Minafra I, Di Liegro I (2003). Purification by affinity chromatography of H1o RNA-binding proteins from rat brain. Int J Mol Med.

[10] Gerstberger S, Hafner M, Tuschl T (2014). A census of human RNA-binding proteins. Nat Rev Genet.

[11] Galante PA, Sandhu D, de Sousa AR, Gradassi M, Slager N, Vogel C, de Souza SJ, Penalva LO (2009). A comprehensive in silico expression analysis of RNA binding proteins in normal and tumor tissue: Identification of potential players in tumor formation. RNA Biol.

[12] Ho JC, Cheung ST, Poon WS, Lee YT, Ng IO, Fan ST (2007). Down-regulation of retinol binding protein 5 is associated with aggressive tumor features in hepatocellular carcinoma. J Cancer Res Clin Oncol.

[13] Newman R, McHugh J, Turner M (2016). RNA binding proteins as regulators of immune cell biology. Clin Exp Immunol.

[14] Zhang BH, Yang J, Jiang L, Lyu T, Kong LX, Tan YF, Li B, Zhu YF, Xi AY, Xu X (2020). Development and validation of a 14-gene signature for prognosis prediction in hepatocellular carcinoma. Genomics.

[15] Chen W, Ou M, Tang D, Dai Y, Du W (2020). Identification and validation of immune-related gene prognostic signature for hepatocellular carcinoma. J Immunol Res.

[16] Jiang L, Zhao L, Bi J, Guan Q, Qi A, Wei Q, He M, Wei M, Zhao L (2019). Glycolysis gene expression profilings screen for prognostic risk signature of hepatocellular carcinoma. Aging.

[17] Shi YM, Li YY, Lin JY, Zheng L, Zhu YM, Huang J (2018). The discovery of a novel eight-mRNA-lncRNA signature predicting survival of hepatocellular carcinoma patients. J Cell Biochem.

[18] Wang Z, Teng D, Li Y, Hu Z, Liu L, Zheng H (2018). A six-gene-based prognostic signature for hepatocellular carcinoma overall survival prediction. Life Sci.

[19] Yan Y, Lu Y, Mao K, Zhang M, Liu H, Zhou Q, Lin J, Zhang J, Wang J, Xiao Z (2019). Identification and validation of a prognostic four-genes signature for hepatocellular carcinoma: integrated ceRNA network analysis. Hep Intl.

[20] Liu GM, Zeng HD, Zhang CY, Xu JW (2019). Identification of a six-gene signature predicting overall survival for hepatocellular carcinoma. Cancer Cell Int.

[21] Li N, Zhao L, Guo C, Liu C, Liu Y (2019). Identification of a novel DNA repair-related prognostic signature predicting survival of patients with hepatocellular carcinoma. Cancer Manage Res.

[22] Heinäniemi M, Nykter M, Kramer R, Wienecke-Baldacchino A, Sinkkonen L, Zhou JX, Kreisberg R, Kauffman SA, Huang S, Shmulevich I (2013). Gene-pair expression signatures reveal lineage control. Nat Methods.

[23] Li B, Cui Y, Diehn M, Li R (2017). Development and validation of an individualized immune prognostic signature in early-stage nonsquamous non-small cell lung cancer. JAMA Oncol.

[24] Li B, Feng W, Luo O, Xu T, Cao Y, Wu H, Yu D, Ding Y (2017). Development and validation of a three-gene prognostic signature for patients with hepatocellular carcinoma. Sci Rep.

[25] Zhao QJ, Zhang J, Xu L, Liu FF (2018). Identification of a five-long non-coding RNA signature to improve the prognosis prediction for patients with hepatocellular carcinoma. World J Gastroenterol.

[26] Chen PF, Li QH, Zeng LR, Yang XY, Peng PL, He JH, Fan B (2019). A 4-gene prognostic signature predicting survival in hepatocellular carcinoma. J Cell Biochem.

[27] Wang Y, Ruan Z, Yu S, Tian T, Liang X, Jing L, Li W, Wang X, Xiang L, Claret FX (2019). A four-methylated mRNA signature-based risk score system predicts survival in patients with hepatocellular carcinoma. Aging.

[28] Qiao GJ, Chen L, Wu JC, Li ZR (2019). Identification of an eight-gene signature for survival prediction for patients with hepatocellular carcinoma based on integrated bioinformatics analysis. PeerJ.

[29] Zhang BH, Yang J, Jiang L, Lyu T, Kong LX, Tan YF, Li B, Zhu YF, Xi AY, Xu X (2020). Development and validation of a 14-gene signature for prognosis prediction in hepatocellular carcinoma. Genomics.

[30] Quattrone A, Dassi E (2019). The architecture of the human RNA-binding protein regulatory network. iScience.

[31] Pereira B, Billaud M, Almeida R (2017). RNA-binding proteins in cancer: old players and new actors. Trends Cancer.

[32] Kosti A, de Araujo PR, Li WQ, Guardia GDA, Chiou J, Yi C, Ray D, Meliso F, Li YM, Delambre T (2020). The RNA-binding protein SERBP1 functions as a novel oncogenic factor in glioblastoma by bridging cancer metabolism and epigenetic regulation. Genome Biol.

[33] Wang H, Huang R, Guo W, Qin X, Yang Z, Yuan Z, Wei Y, Mo C, Zeng Z, Luo J (2020). RNA-binding protein CELF1 enhances cell migration, invasion, and chemoresistance by targeting ETS2 in colorectal cancer. Clin Sci.

[34] Wang H, Sun Q, Zhao W, Qi L, Gu Y, Li P, Zhang M, Li Y, Liu SL, Guo Z (2015). Individual-level analysis of differential expression of genes and pathways for personalized medicine. Bioinformatics (Oxford, England).

[35] Eddy JA, Sung J, Geman D, Price ND (2010). Relative expression analysis for molecular cancer diagnosis and prognosis. Technol Cancer Res Treat.

[36] Masuda K, Kuwano Y (2019). Diverse roles of RNA-binding proteins in cancer traits and their implications in gastrointestinal cancers. Wiley Interdiscipl Rev RNA.

[37] Abdelmohsen K, Srikantan S, Kuwano Y, Gorospe M (2008). miR-519 reduces cell proliferation by lowering RNA-binding protein HuR levels. Proc Natl Acad Sci USA.

[38] Guo X, Wu Y, Hartley RS (2009). MicroRNA-125a represses cell growth by targeting HuR in breast cancer. RNA Biol.

[39] Goudarzi KM, Lindström MS (2016). Role of ribosomal protein mutations in tumor development (Review). Int J Oncol.

[40] Wang T, Xu L, Jia R, Wei J (2017). MiR-218 suppresses the metastasis and EMT of HCC cells via targeting SERBP1. Acta Biochim Biophys Sin.

[41] Zhao M, Jia M, Xiang Y, Zeng Y, Yu W, Xiao B, Dai R (2020). METTL3 promotes the progression of hepatocellular carcinoma through m(6)A-mediated up-regulation of microRNA-873–5p. Am J Physiol Gastrointest Liver Physiol.

[42] Wang ZL, Li B, Luo YX, Lin Q, Liu SR, Zhang XQ, Zhou H, Yang JH, Qu LH (2018). Comprehensive genomic characterization of RNA-binding proteins across human cancers. Cell Rep.

[43] Guinney J, Dienstmann R, Wang X, de Reyniès A, Schlicker A, Soneson C, Marisa L, Roepman P, Nyamundanda G, Angelino P (2015). The consensus molecular subtypes of colorectal cancer. Nat Med.

[44] Zhao E, Zhou C, Chen S (2020). A signature of 14 immune-related gene pairs predicts overall survival in gastric cancer. Clin Transl Oncol.

[45] Zhou Y, Zhou B, Pache L, Chang M, Khodabakhshi AH, Tanaseichuk O, Benner C, Chanda SK (2019). Metascape provides a biologist-oriented resource for the analysis of systems-level datasets. Nat Commun.

